# Inositol hexakisphosphate kinase-1 is a key mediator of prepulse inhibition and short-term fear memory

**DOI:** 10.1186/s13041-020-00615-3

**Published:** 2020-05-07

**Authors:** Min-Gyu Kim, Seungjae Zhang, Hoyong Park, Seung Ju Park, Seyun Kim, ChiHye Chung

**Affiliations:** 1 Department of Biological Sciences, Korea Advanced Institute of Science and Technology (KAIST), Daejeon, 34141 Korea; 2 Department of Biological Sciences, Konkuk University, Seoul, 05029 Korea; 3 KAIST Institute for the BioCentury, KAIST, Daejeon, 34141 Korea

**Keywords:** Inositol pyrophosphate, IP6K1, Prepulse inhibition, Short-term memory

## Abstract

Inositol phosphate metabolism has emerged as one of the key players in synaptic transmission. Previous studies have shown that the deletion of inositol hexakisphosphate kinase 1 (IP6K1), which is responsible for inositol pyrophosphate biosynthesis, alters probability of presynaptic vesicle release and short-term facilitation of glutamatergic synapses in mouse hippocampus. However, the behavioral and cognitive functions regulated by IP6K1 remain largely elusive. In this study, IP6K1-knockout mice exhibited decreased prepulse inhibition with no defects in Y-maze and elevated plus maze tests. Interestingly, IP6K1 knockout led to impaired short-term memory formation in a contextual fear memory retrieval test with no effect on long-term memory. Further, both hippocampal long-term potentiation and long-term depression in IP6K1-knockout mice were similar to those in the wild-type control. Taken together, the findings in this study suggest the physiological roles of IP6K1 and the associated inositol pyrophosphate metabolism in regulating sensorimotor gating as well as short-term memory.

## Main text

Hydrolysis of phosphatidylinositol 4,5-bisphosphate by phospholipase C produces inositol 1,4,5-trisphosphate (IP_3_), which triggers cytosolic calcium release. IP_3_ becomes metabolized into highly phosphorylated inositol phosphates (IPs) through the actions of IP kinases [1]. IPs and their metabolic enzymes seem to play critical roles in the brain [2, 3]. Inositol pyrophosphates are unique among the IPs in that they contain high-energy diphosphates. The most prominent inositol pyrophosphate is 5-diphosphoinositol pentakisphosphate (5-IP_7_) [1, 4], which inhibits Akt signaling in various tissues, including brain [1, 2]. 5-IP_7_ is generated by inositol hexakisphosphate kinases (IP6Ks), which add a phosphate in the 5-position of inositol hexakisphosphate (IP_6_) [1, 4].

Among the three IP6K isoforms (IP6K1/2/3), IP6K1 has the highest expression levels in the brain [2]. IP6K1 and its product 5-IP_7_ appear to facilitate glycogen synthase kinase (GSK) signaling in the mouse brain. IP6K1-knockout (IP6K1-KO) mice further showed decreased locomotor activity in response to amphetamine [2]. Recently, IP6K1’s product, 5-IP_7_, was shown to bind and suppress synaptotagmin 1 (Syt1), a presynaptic Ca^2+^ sensor, thus inhibiting synaptic vesicle exocytosis [5]. Park et al., reported increased presynaptic release probability, decreased short-term facilitation, and impaired presynaptic endocytosis from IP6K1-KO hippocampal neurons [6]. Dysfunction of synaptic vesicle cycling has been linked to a number of behavior deficits including a neurodevelopmental mental illness, schizophrenia [7, 8]. Some schizophrenia patients exhibit altered expression or mutations of proteins related to presynaptic vesicle release such as synapsins [8]. Considering pivotal roles of IP6K1 and 5-IP_7_ in the control of presynaptic vesicle cycling and Akt-GSK signaling in the brain, we wondered about the impact of IP6K1-knockout in behavior. Whether inositol pyrophosphate metabolism regulates learning and memory remains largely unknown.

**Fig. 1:**
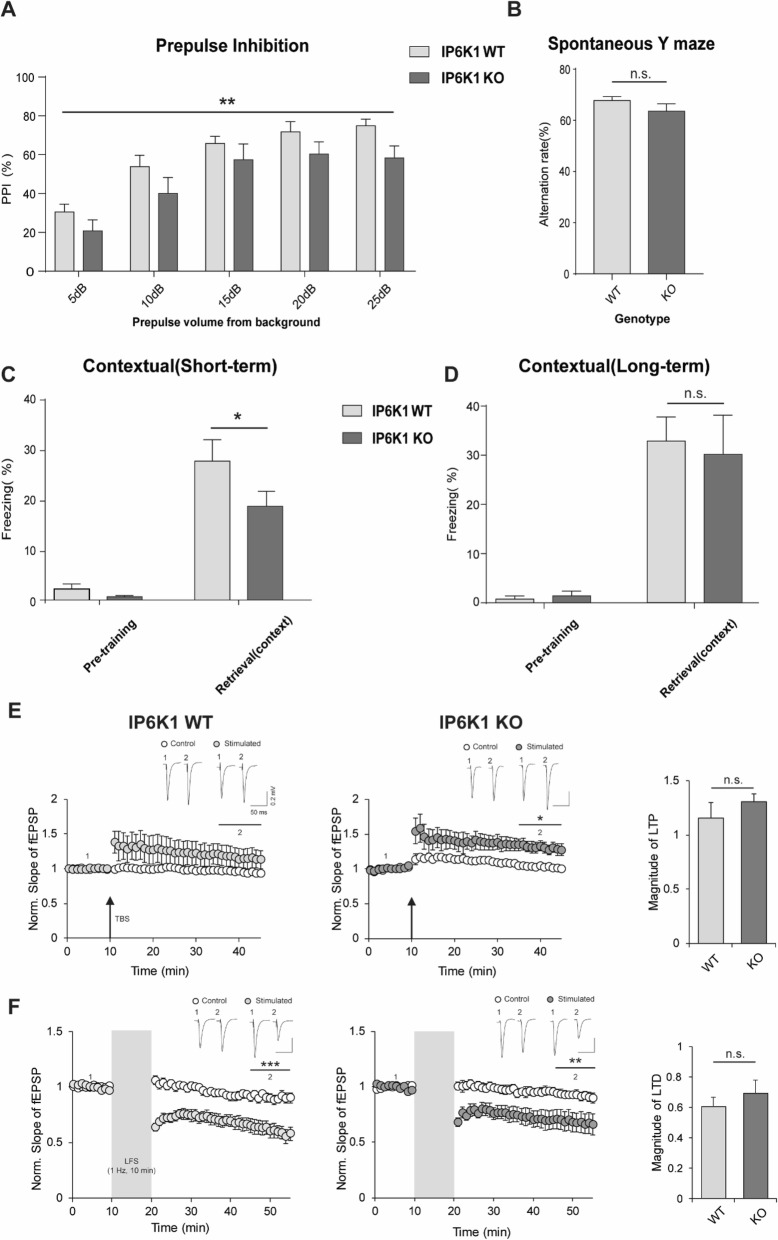
IP6K1-KO mice show schizophrenia-like behavior and impaired hippocampus-dependent short-term memory but normal in long-term memory. **a** Significant decrease in PPI of acoustic startle in IP6K1 KO mice compared to IP6K1 WT littermate controls around all levels of prepulse (WT: *n* = 7; KO: *n* = 6; two-way ANOVA; main effect of genotype, F_(1,55)_ = 11.03, **P < 0.01; main effect of prepulse level, F_(4,55)_ = 18.79, *P* < 0.0001;no significant interaction). **b** No change in Spontaneous Y maze at alteration rate in IP6K1 KO mice versus IP6K1 WT littermate controls(WT: *n* = 6; KO: *n* = 8; two tailed student t-test; ns: no significant; *P* ≥ 0.05). **c** Freezing levels of IP6K1 WT and IP6K1 KO mice before (pretraining) and 1 h after (retrieval) contextual fear shock. IP6K1 KO mice exhibited significantly decreased freezing levels during retrieval test versus IP6K1 WT (WT: *n* = 13; KO: *n* = 14; two-way ANOVA; main effect of genotype, F_(1,50)_ = 4.04, *P < 0.05; main effect of condition, F_(1,50)_ = 68.45, *P* < 0.0001; Bonferroni posttests, *P < 0.05). **d** Freezing levels of IP6K1 WT and IP6K1 KO mice before (pretraining) and 24 h after (retrieval) contextual fear conditioning. IP6K1 KO mice show no significant freezing level change compared to IP6K1 WT mice (WT: *n* = 7; KO: *n* = 6; two-way ANOVA; main effect of genotype, F_(1,22)_ = 0.05, *P* ≥ 0.05; main effect of condition F_(1,22)_ = 44.84, *P* < 0.0001; Bonferroni posttest, ns: not significant). **e** Hippocampal LTP is occurred on IP6K1 KO (WT: *n* = 5; paired t-test; potentiation, 1.15 ± 0.14, *P* ≥ 0.05; KO: *n* = 6; paired t-test; potentiation, 1.30 ± 0.07, *P < 0.05), but not significantly difference on magnitude of LTP. (paired t-test; *P* ≥ 0.05; ns: no significant). **f** LFS-induced hippocampal LTD is occurred both IP6K1 WT and IP6K1 KO (WT: *n* = 7; paired t-test; depression: 0.60 ± 0.05; ***P < 0.001; KO: *n* = 7; paired t-test; depression, 0.69 ± 0.02, **P < 0.01). Magnitude of LTD were no significant change between IP6K1 WT and IP6K1 KO. (paired t-test; *P* ≥ 0.05; ns: no significant)

We performed behavioral analyses to determine whether IP6K1-KO mice show any changes in sensorimotor gating, one of the well-known characteristics of schizophrenia. IP6K1-KO mice exhibited lower prepulse inhibition (PPI) values than wild-type (WT) mice (Fig. 1a). In order to examine the effect of IP6K1 deficiency on spatial working memory, we next tested Y-maze spontaneous alternation and found no significant difference in the alteration rate between control and IP6K1-KO mice (Fig. 1b). When the animals were tested in an elevated plus maze experiment, IP6K1-WT and IP6K1-KO mice showed no difference in the time spent on each arm (Additional file 1; Sup Fig.1). These findings clearly suggest that IP6K1 plays a role in the control of sensorimotor gating.

Since IP6K1-KO mice showed defects in synaptic vesicle trafficking in the hippocampus, we next focused on the hippocampus-dependent associative fear memory to determine whether IP6K1 influences memory processes. Both groups of mice were trained by administering a single electric foot shock (0.6 mA, 2 s) in a specific chamber for contextual fear conditioning. Prior to electric shock exposure, the freezing rate of IP6K1-KO mice was similar to that of IP6K1-WT mice. After training, the mice were re-exposed to the same chamber after a delay of 1 and 24 h, and the freezing rate was measured. Under the short-term memory paradigm, re-exposure to the conditioned context with a 1-h delay revealed a lower freezing rate in IP6K1-KO mice (Fig. 1c), suggesting impaired short-term memory following the deletion of IP6K1. In contrast, when exposure to the same context 24 h after training revealed no difference in the freezing rate between IP6K1-WT and IP6K1-KO mice (Fig. 1d). These findings indicate a selective action of IP6K1 in the control of short-term memory.

To further investigate the impact of IP6K1 deficiency in synaptic plasticity, extracellular recording was performed in acute brain slices. LTP was induced using theta burst stimulation (TBS), a well-known and physiologically relevant stimulation protocol. TBS successfully induced LTP both in WT and IP6K1-KO mice and the levels of potentiation were comparable between groups (Fig. 1e). When low-frequency stimulation (LFS) was performed to induce long-term depression (LTD), LTD was intact in both WT and IP6K1-KO mice and the magnitudes of LTD were not different in both groups (Fig. 1f). These observations suggest that long-term synaptic plasticity remains largely intact even in the absence of IP6K1, implying that IP6K1 is dispensable for the induction and maintenance of long-term synaptic plasticity.

In this study, we identified the roles of IP6K1 in behaviors. The results suggested that IP6K1 and 5-IP_7_ are physiological determinants underlying PPI and short-term memory. PPI, a measure of sensorimotor gating during a short interval (50–200 ms) of prepulse and the startle pulse, is decreased in schizophrenic patients. Key factors for controlling PPI include presynaptic proteins such as Rab3A and Syt1 that are known to regulate short-term plasticity in the order of 50–200 ms [9]. The Rab3A deletion or Syt1 mutation caused increased paired-pulse facilitation accompanied with lowered PPI [9]. IP6K1 is known to regulate Rab3A activity by modulating its interaction with GRAB [10] and 5-IP_7_ is shown to suppress Syt1 functions in synaptic exocytosis [5]. Therefore, given the role of IP6K1 and 5-IP_7_ in controlling presynaptic vesicle cycling [5, 6, 10] and Akt-GSK signaling [2], IP6K1 deletion is likely to alter the PPI. Both Akt1 knockout mice [11] and prefrontal cortex-specific GSK3β inhibition [12] indeed exhibited impaired PPI.

Recently, marked changes in short-term synaptic plasticity were found in IP6K1-KO hippocampal neurons [6]. Interestingly, IP6K1 deletion led to selective reduction in short-term memory with preserved long-term memory as well as normal synaptic plasticity in LTP and LTD. Thus, the actions of 5-IP_7_ appear effective for only a short period. As synaptic vesicle recycling is a critical event at synapses to maintain information transfer, it is possible that IP6K1-KO synapses may operate protective systems to prevent long-term failure of information processing.

Short-term and long-term memory processes are tightly linked, but the two processes are clearly distinct. As shown in IP6K1-KO mice, short-term but not long-term memory was selectively impaired in the mouse model of Down syndrome [13] and NF-κB deletion [14]. Given that dentate gyrus specific GSK3β disruption caused impaired contextual fear memory [15], IP6K1-GSK3β pathway may be responsible for lowered PPI and impaired short-term memory formation. Future studies will elucidate the details of how IP6K1 and inositol pyrophosphates contribute to the control of short-term memory formation, storage, and retrieval and how short-term memory deficit and sensorimotor gating delay are intertwined at circuit levels. Our findings that IP6K1, as a physiological player, regulates behavior highlight the significance of inositol pyrophosphate metabolism in the brain and provide insights into the treatment and management of psychiatric diseases such as schizophrenia.

## Supplementary information

**Additional file 1:Figure S1.** The anxiety level in IP6K1-KO mice did not show difference compared with WT mice. (WT *n* = 7; KO *n* = 7; two-way ANOVA; main effect of genotype, F_(1,24)_ = 7.898Xe^-10^, *P* > 0.9999, effect of position, F_(1,24)_ = 72.34, *P* < 0.0001; Bonferroni posttest, ns: not significant).

**Additional file 2.** Materials and Methods.

## Data Availability

All authors agree to share data and materials upon request.
