# Supplementary material for: Inositol hexakisphosphate kinase-1 is a key mediator of prepulse inhibition and short-term fear memory
Source: Mol Brain. 2020 May 7;13:72. doi: 10.1186/s13041-020-00615-3 (PMC7206715; doi:10.1186/s13041-020-00615-3)
Supplement: Supplementary file 1 — Additional file 1:Figure S1. The anxiety level in IP6K1-KO mice did not show difference compared with WT mice. (WT n = 7; KO n = 7; two-way ANOVA; main effect of genotype, F(1,24) = 7.898Xe-10, P > 0.9999, effect of position, F(1,24) = 72.34, P < 0.0001; Bonferroni posttest, ns: not significant). [file 13041_2020_615_MOESM1_ESM.pdf]

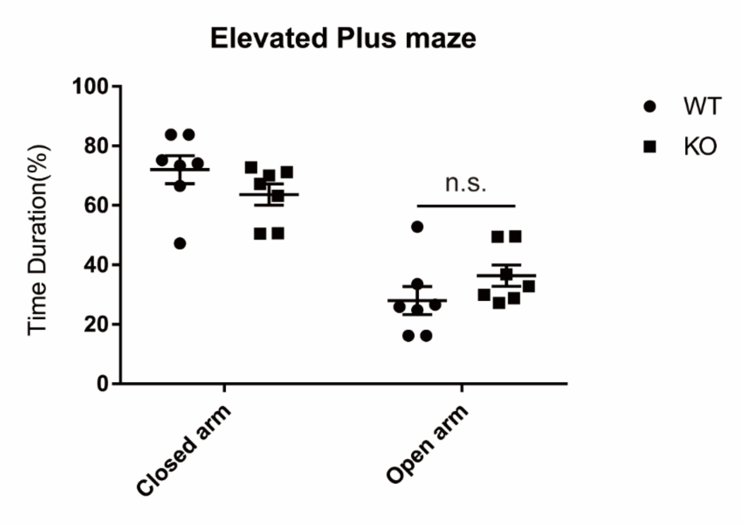

Supplementary Figure 1. The anxiety level in IP6K1-KO mice did not show difference compared with WT mice. (WT n = 7; KO n = 7; two-way ANOVA; main effect of genotype, $F_{(1,24)} = 7.898Xe^{-10}$, $P > 0.9999$, effect of position, $F_{(1,24)} = 72.34$, $P < 0.0001$; Bonferroni posttest, ns: not significant)
