# Supplementary material for: Inositol hexakisphosphate kinase-1 is a key mediator of prepulse inhibition and short-term fear memory
Source: Mol Brain. 2020 May 7;13:72. doi: 10.1186/s13041-020-00615-3 (PMC7206715; doi:10.1186/s13041-020-00615-3)
Supplement: Supplementary file 2 — Additional file 2. Materials and Methods. [file 13041_2020_615_MOESM2_ESM.pdf]

Materials and Methods

Mice

Whole body IP6K1 knockout mice were generated by Cas9/CRISPR method, as described previously [1]. This mouse line was back-crossed with the C57BL/6J mouse more than six times to remove the off target. Briefly, all IP6K1-KO mice used for experiment with their littermates, IP6K1-WT mice, which are controls. Animals were bred and housed under specific pathogen-free environment in a 12 h light-dark schedule, and their water and food were supplied ad libitum. Mice used for all behavioral experiments were handled for 5-consecutive days for 2 min before conducting behavioral experiments. Animal protocols were performed in accordance with guidelines approved by the Korea Advanced Institute of Science and Technology Animal Care and Use Committee.

Prepulse inhibition

The experimental mouse was placed in a startle chamber (SR-LAB). For adaptation, a background noise of 65 dB pulse was given for 5 min. After adaptation, 57 testing sound pulses with random inter-trial intervals (7-23 sec) were given. The composition of testing sound pulses were 4 pulses (4 x 120 ms, 120 dB) in the beginning and end stage of the test, and 7 pulses (120 ms, 120 dB each) paired with prepulses (20 ms – 100 ms prior to) at 70, 75, 80, 85 and 90 dB (total 35 paired pre-pulses). Rate [%] of prepulse inhibition was calculated by following formula, PPI [%] = 100 x (startle reactivity – reactivity on prepulse-pulse trials)/startle reactivity

Spontaneous alternation in Y-maze test

The apparatus consisted of three-arm with equal angles between all arms (30 x 5 x 12 cm). Mice placed within one arm were allowed to free explore all three arms of the Y-maze for 8 min. The sequence of arm entries was recorded automatically and spontaneous alternation of last 5 min is calculated manually. Spontaneous alternation [%] was defined as consecutive entries in 3 different arms (ABC, BAC, or CAB), divided by number of possible alternations (total entries -2).

Elevated plus maze

The composition of elevated plus maze were two open arms, two closed arms, and a center zone. This maze elevated to a height of 50 cm above the floor. Mice were placed in the center zone and allowed to explore the maze for 5 min. The data was analyzed using EthoVision 3.1 (Noldus).

Contextual fear memory retrieval test

The mice experienced a single electrical foot shock (0.6 mA for 2 sec) after being in the fear chamber for 3 minutes. Then, mice were returned to the cages. For fear memory retrieval test, mice were placed in the same chamber and exposed to the same context for 3 min at 1 h or 24 h after shock. Freezing (immobile posture except for respiration) level was measured automatically by using a computer program (Freeze Frame3, Coulbourn Instruments).

Slice preparation and electrophysiology

IP6K1 KO mice and IP6K1 WT littermates (8-10 weeks old) were used for electrophysiological experiment. All animals were anesthetized by using isoflurane, and immediately decapitated. Brains were stored cold choline dissection buffer (containing in mM: 110 choline chloride, 11.6 Na-ascorbate, 3.1 pyruvate, 25 $NaHCO_3$, 1.25 NaH_2PO_4, 2.5 KCl, 7 $MgCl_2$, 0.5 $CaCl_2$ and

25 glucose, bubbled with 95% O_2 and 5% CO_2). Coronal slices (350 μm thick) were prepared using a vibratome (VT 1000s, Leica) and allowed to rest in artificial cerebrospinal fluid (aCSF, containing in mM: 1 NaH_2PO_4, 26.2 $NaHCO_3$, 118 NaCl, 2.5 KCl, 11 glucose, 2 $CaCl_2$ and 1 $MgCl_2$, bubbling with 95% O_2 and 5% CO_2) containing chamber at 35°C water bath for 45 min. Slices were stored at room temperature and then transferred to the recording chamber while constantly perfused aCSF at 30 - 32°C.

Field excitatory postsynaptic potential (fEPSP) recordings were obtained using a DAM 80 and WinLTP 2.10 (University of Bristol), filtered 3 kHz and sampled at 20 kHz. Recording pipettes with resistance of 1-3 MΩ were filled with aCSF. fEPSPs were recorded from the Schaffer collateral pathway (CA3-to-CA1 synapses). Electrical stimulation used two bipolar electric stimulators (FHC) placed on stratum radiatum, alternatively stimulate by 20 sec inter-stimulation interval (ISI). Long-term potentiation (LTP) experiment obtained by using theta burst stimulation (TBS) composing one stimulus train of 100 Hz (5 pulses) 20 times at 5 Hz intervals. Long-term depression (LTD) was induced by low frequency stimulation (LFS) at 1 Hz (600 pulses).

Statistical analysis

Differences between averages were analyzed using a two-tailed Student's *t* test or two-way ANOVA followed by Bonferroni's posttest. Electrophysiological experiments were analyzed by comparing the baseline (averaged for first 10 min) and the mean value of 25 min after the stimulation using paired *t*-test. All data were presented as mean±SEM. Statistical significance was set at $P < 0.05$.

Supplementary reference

1. Park SJ, Park H, Kim MG, Zhang S, Park SE, Kim S, et al. Inositol pyrophosphate metabolism regulates presynaptic vesicle cycling at central synapses. iScience. 2020; 23(4): 101000.
